# Intersection of Inflammation and Senescence in the Aging Lung Stem Cell Niche

**DOI:** 10.3389/fcell.2022.932723

**Published:** 2022-07-13

**Authors:** Nancy C. Allen, Nabora S. Reyes, Jin Young Lee, Tien Peng

**Affiliations:** ^1^ Department of Medicine and Division of Pulmonary, Critical Care, Allergy and Sleep Medicine, University of California, San Francisco, San Francisco, CA, United States; ^2^ Bakar Aging Research Institute, University of California, San Francisco, San Francisco, CA, United States

**Keywords:** senescence, stem cell niche, SASP, inflammation, aging

## Abstract

Aging is the final stage of development with stereotyped changes in tissue morphology. These age-related changes are risk factors for a multitude of chronic lung diseases, transcending the diverse pathogenic mechanisms that have been studied in disease-specific contexts. Two of the hallmarks of aging include inflammation and cellular senescence, which have been attributed as drivers of age-related organ decline. While these two age-related processes are often studied independently in the same tissue, there appears to be a reciprocal relationship between inflammation and senescence, which remodels the aging tissue architecture to increase susceptibility to chronic diseases. This review will attempt to address the “chicken or the egg” question as to whether senescence drives inflammation in the aging lung, or vice versa, and whether the causality of this relationship has therapeutic implications for age-related lung diseases.

## Introduction

While initial large increases in life expectancy during the first half of the 20th century are attributable to improved sanitation and the introduction of antibiotics, a slower rise has occurred over the last 50 years due to a gradual decline in deaths from noncommunicable diseases of aging, such as cancer and cardiovascular disease (Crimmins and Zhang, 2019). As the population ages, diseases of the lungs are becoming a particularly important contributor to morbidity and mortality. In 2019 the World Health Organization (WHO) reported that chronic obstructive pulmonary disease (COPD), lower respiratory tract infections, and cancers of the respiratory tract accounted for three of the top ten causes of death in the world, with deaths from COPD only outnumbered by stroke and ischemic heart disease (www.who.int). In 2020, COVID-19, which has disproportionately impacted the elderly, became the third leading cause of death in the United States (https://www.cdc.gov). With these statistics in mind, it has become clear that improved understanding of physiologic aging and how aging increases susceptibility to lung disease are critical for optimizing population health.

Aging is a complex physiologic process that can be conceptualized as an age-dependent impairment in the maintenance of tissue homeostasis, ultimately leading to loss of optimal tissue function (López-Otín et al., 2013). In addition to impaired maintenance of homeostasis, an additional feature of aging is deterioration of the cellular and physiologic response to stress, resulting in worse outcomes for the same injury in an aged versus young organism (Lipsitz and Goldberger, 1992). Cellular senescence, a coordinated cellular response to stress characterized by permanent cell cycle exit and the development of an elaborate secretory profile, is intricately linked with aging. It is well-appreciated that the number of senescent cells increases with age, and the removal of senescent cells through various mechanisms has been shown to improve both healthspan and median lifespan in mice (Baker et al., 2011; Baker et al., 2016; Tabula Muris, 2020). The senescent cell secretory profile, commonly referred to as the senescence-associated secretory phenotype (SASP), is considered one of the major mechanisms by which senescent cells impact their resident tissues. The SASP—which frequently encompasses cytokines, chemokines, and growth factors—is thought to mediate its effects through multiple mechanisms, including direct action on tissue-resident stem cells and immune cell recruitment (Janzen et al., 2006; Krishnamurthy et al., 2006; Kang et al., 2011; Kulkarni et al., 2019; Sturmlechner et al., 2021).

The human lung has a surface area of approximately 70 m^2^, with an elaborate epithelial structure to accomplish its numerous functions, which include mucus production and clearance, antimicrobial defense, surfactant production, and the facilitation of gas exchange (Fröhlich et al., 2016; Leiva-Juárez et al., 2018). Maintenance and repair of the epithelium requires proper functioning of airway epithelial stem cells (Basil et al., 2020). In the conducting airways, the major stem cells are basal and secretory club cells, while in the alveoli which are involved in gas exchange the predominant progenitor is the alveolar type 2 (AT2) cell (Barkauskas et al., 2013; Davis and Wypych, 2021). These stem cells are both supported by and responsive to signals from their niche cells, which usually include, but are not limited to fibroblasts, endothelial, and resident immune cells (Donne et al., 2015; Peng et al., 2015; Lechner et al., 2017; Zepp et al., 2017). Emerging data have demonstrated that cells of the lung stem cell niche can express cytokines and growth factors that overlap with SASP factors, and that these secreted factors can alter stem cell behavior, thus offering a potential mechanism through which the aging niche impacts stem cell function.

Here we explore the mechanisms by which senescent cells develop in the aging lung, and how these cells contribute to both physiologic aging and aging-associated lung diseases. We give particular attention to mechanisms by which senescent cells interact with the lung stem cell niche, and how senescent cell interaction with the immune system can modulate not only tissue immune cell composition but also immune cell function. Finally, we explore the potential contribution of senescence to the pathogenesis of some of the most common age-related diseases in the lung, highlighting the therapeutic implications of unraveling the intersection between senescence and inflammation in the aging lung.

## Cellular Senescence and Its Functions

Cellular senescence is a multifaceted cell state that is classically characterized by permanent cell cycle exit (Salama et al., 2014). The first described example of senescence was replicative senescence, referring to the finite number of cell divisions allowed for a normal cell. However, it is now well-appreciated that senescence can develop in response to a number of cellular stresses, including oncogene activation, DNA damage, or reactive oxygen species (ROS) (Hayflick and Moorhead, 1961; Hayflick, 1965; Campisi and d'Adda di Fagagna, 2007). The two main pathways that activate senescence in response to cellular stress are the p53-p21 axis and p16^INK4a^ (aka p16). Both p21 and p16^INK4a^ are inhibitors of cyclin-dependent kinases (CDKs), and ultimately function to prevent the inactivation of RB and subsequent cell cycle entry (van Deursen, 2014). In addition to p53 and p16^INK4a^ expression, other common features of senescent cells include an enlarged, flat morphology, senescence-associated beta-galactosidase (SA-B-gal) activity, senescence-associated heterochromatic foci (SAHF), and a SASP (Salama et al., 2014). The SASP, as discussed in more detail later, plays a central role in maintenance of the senescent phenotype and the effector functions of senescent cells. While there is no single unique marker of senescence, p16^INK4a^ expression, encoded by *Cdkn2a*, is considered to be one of the most specific features, and is frequently used in studies to identify and manipulate senescent populations (Rodier et al., 2009; Grosse et al., 2020; Omori et al., 2020). Nonetheless, given the lack of a single specific marker, an integrated approach that incorporates phenotypic, molecular, and functional data is likely needed for the most accurate identification of cellular senescence.

While senescence has been found to play a role in multiple contexts, including developmental patterning and wound healing, it has most widely been appreciated as a mechanism by which damaged cells exit the cell cycle to avoid cancer formation (Campisi, 2001; Muñoz-Espín et al., 2013; Storer et al., 2013). Despite the supposed anti-tumor benefits of senescence, there has been considerable effort within the scientific community to find methods to remove senescent cells. These efforts are motivated by recent studies demonstrating that the genetic deletion of p16^INK4a^ expressing cells extends median lifespan and slows many aging-associated disorders in mice, including but not limited to renal dysfunction, cardiac arrhythmogenesis, cataract formation, decreased adipose reserve, and declines in spontaneous activity (Baker et al., 2011; Baker et al., 2016; Baar et al., 2017). Indeed, the number of senescent cells increases with age, and their contribution to aging has been linked to impaired stem cell renewal, diminished cellular regeneration, and the development of a pro-inflammatory environment that further impairs tissue function (Janzen et al., 2006; Krishnamurthy et al., 2006; Tilstra et al., 2012; Tabula Muris, 2020).

There are several theories to explain these seemingly contradictory roles for senescent cells. The most commonly cited theory is that of antagonistic pleiotropy. This theory argues that while senescence is beneficial in youth, its detrimental functions in aging persist due to a lack of selection pressure late in life (Campisi, 2016). Support for this theory comes from recent genome-wide association studies identifying single nucleotide polymorphisms (SNPs) that are protective against youth-associated diseases but increase risk for diseases that tend to arise later in life. Interestingly, one SNP identified in this study was in the *CDKN2A* locus, which is protective against glioma but increases the risk of later-onset diseases such as glaucoma, type 2 diabetes, and coronary artery disease (Rodríguez et al., 2017). An alternative theory is one of evolutionary cost and benefit, which argues that while senescence has a beneficial function throughout life, the cost of senescence outweighs the benefits with advancing age (Di Micco et al., 2021). Certainly, these theories are not mutually exclusive, and it seems likely that within the complex senescence phenotype certain characteristics align with different selection paradigms.

## Inflammation and SASP as a Major Component of Senescence

Far from being quiescent, senescent cells are metabolically active and constantly interacting with their environment through the secretion of a complex secretory profile known as the SASP. One problematic feature of defining the SASP is that it encompasses diverse cytokines, chemokines, growth factors, and extracellular matrix modifiers that are often dependent on both cell type and method of senescence induction (Di Micco et al., 2021). Despite there being considerable heterogeneity among SASPs, one common feature is the ability of the SASP to induce local inflammation through mediators such as IL-6, IL-8, and PAI-1 (Kortlever et al., 2006; Salama et al., 2014).

An inflammatory response, which is frequently mediated by the SASP, turns out to be critical for both the initiation and maintenance of senescence, as well as important senescent cell effector functions such as the induction of immunosurveillance (Acosta et al., 2008; Kuilman et al., 2008; Sturmlechner et al., 2021). Interestingly, a persistent DNA damage response (DDR) has been shown to be important for induction of the core SASP factors IL-6 and IL-8 in senescence, whereas a transient DDR or *p16*-overexpression without coincident DDR failed to induce these genes, reinforcing the role of senescence in preventing tumorigenesis (Rodier et al., 2009). Indeed, the induction of senescence has been particularly well-studied in the setting of oncogene-induced senescence (OIS). In this context, oncogene-induced stress increases the transcription of *CEBPB*, which leads to the upregulation of IL-6, IL-8, and other SASP proteins. Autocrine IL-6 signaling, in particular, was identified as an important regulator of both OIS induction and maintenance (Kuilman et al., 2008). IL-8 has also been shown to play a role in the induction and maintenance of both replicative and OIS via autocrine signaling through CXCR2. In this study, using MEK1 activation to induce OIS, both C/EBPb and NF-kB were shown to play important roles in the induction of IL-8 and other SASP genes (Acosta et al., 2008).

It is becoming increasingly evident that NF-kB, a master regulator of inflammatory responses, plays a central role in regulating the SASP. *In vitro*, p65, the main activating subunit of NF-kB, has been found to accumulate on chromatin during the development of senescence, and knock-down of *p65* severely impairs the SASP without reversing cell-cycle arrest (Chien et al., 2011). Similar evidence *in vivo* has shown that NF-kB activation increases with age, and genetic deletion of *p65* or p65 inhibition reduces senescence, SASP production, and ultimately the onset of age-associated diseases in progeroid mice (Tilstra et al., 2012). There are several mechanisms by which NF-kB can be activated during senescence induction, of which DNA damage-induced post-translational modifications of NEMO, and stress-induced p38MAPK activation of NF-kB have been well-described. Other proposed mechanisms of NF-kB activation in the setting of senescence include RIG-I-induced inflammasome activation, TGFb-TAK1 signaling, HMGB1 release by stressed or injured cells, and even ceramide accumulation (Miyamoto, 2011; Salminen et al., 2012). Regardless of the mechanism by which NF-kB is initially activated, its activation can initiate a positive feedback loop through the release of NF-kB activating cytokines to help maintain NF-kB activation and the SASP.

In addition to the role inflammatory pathways play in the induction and maintenance of senescence, there is also evidence that senescent cells can utilize inflammation to propagate senescence to nearby cells, a phenomenon called “paracrine senescence”. Evidence for paracrine senescence was demonstrated in a model of OIS in which oncogene-induced senescent cells were able to bring about senescence of nearby normal cells through the secretion of IL-1α, IL-1β, and TGFβ (Acosta et al., 2013). Similarly, in a model of NOTCH1-driven senescence (NIS), senescent cells were able to induce growth arrest and other senescent features in normal neighboring cells. In this case, NIS cell upregulation of TGFb and the NOTCH ligand JAG1 was responsible for the propagation of senescence (Hoare et al., 2016).

An important function attributed to the SASP is the induction of immunosurveillance, referring to the ability of senescent cells to induce an inflammatory response with the presumed objective of early immune cell recognition and removal of cancerous and precancerous cells. The significance of immunosurveillance was demonstrated with a mouse model of OIS, in which the SASP from pre-malignant senescent hepatocytes induced immune cell recruitment and removal of the pre-malignant cells (Kang et al., 2011). The importance of the immune response in this setting was highlighted using SCID mice which lack an adaptive immune system. In this case, the SCID mice were unable to clear the pre-malignant cells and the development of hepatocellular carcinoma was observed (Kang et al., 2011). A recent study dissected the development of senescence and associated immunosurveillance, identifying a mechanism by which *p21* induced early in the development of senescence leads to the upregulation of *Cxcl14* and the recruitment of monocytes and macrophages (Sturmlechner et al., 2021). However, a big caveat for these studies is the proper identification of senescent cells that occur physiologically *in vivo*, as not all cells that express *p21* are senescent. Furthermore, the same paper that described the effect of *p21* induction on immunosurveillance also showed that *p16* induction did not have the same effect. This is consistent with our recent study that demonstrates the presence of a stable, resident *p16+* fibroblast population in the lung over the lifespan (Reyes de Mochel et al., 2020). Regardless of the diversity of immune response to the presence of cells with senescent characteristics, part of the senescence response appears to alert the immune system of their presence.

In the lung, like many tissues, senescent cells accumulate with age (Hashimoto et al., 2016; Lee et al., 2021). There is also some evidence to suggest that senescent cells may develop in younger animals in response to recurrent injury such as intranasal lipopolysaccharide (LPS) or cigarette smoke (Rashid et al., 2018; Sagiv et al., 2018). Therefore, in these contexts, senescent cells can alter their microenvironments through the SASP. It is likely that within the lung NF-kB is an important mediator of the SASP, as it is in most contexts. Potential NF-kB activating stimuli include cell-intrinsic activation in response to DNA damage or other cellular stress, or from cell-extrinsic sources (Salminen et al., 2012). Supporting a role for cell-extrinsic sources of NF-kB activation, analysis of gene expression patterns from aged lungs identifies TNF and IL-1B, both strong NF-kB activating stimuli, as predicted upstream regulators of gene expression (Angelidis et al., 2019). NOTCH signaling is also a potentially interesting regulator of the SASP in the lung, as it has been shown to regulate the SASP profile in OIS, and has also been demonstrated to be an important regulator of lung epithelial stem cell differentiation (Hoare et al., 2016; Choi et al., 2021). Additionally, it is known that the common SASP factor IL-6 is increased in the bronchoalveolar lavage (BAL) of healthy aged individuals (Meyer et al., 1996). It remains to be determined whether the source of IL-6 in this context is from the senescent lung parenchyma or age-related changes in immune system composition or function. Nonetheless, this increase in IL-6 has the potential to both reinforce cellular senescence and impact the local stem cell niche. Below we discuss how common SASP factors have been shown to impact lung stem cell function.

## Impact of Common SASP Factors on Lung Stem Cell Function

### Overview of Lung Structure and Epithelial Stem Cell Niche

The respiratory system can very broadly be delineated into two functional categories: air conduction and gas exchange. The conducting airways include the trachea, bronchi, and conducting bronchioles, of which the epithelium is predominantly composed of secretory and ciliated cells. The primary secretory cell is the club cell, which has multiple functions including the release of the anti-inflammatory protein CCSP, xenobiotic metabolism, and functioning as an airway epithelial progenitor. Ciliary cells, on the other hand, are critical for effective mucus clearance (Davis and Wypych, 2021). In the proximal conducting airways basal cells function as the primary progenitor, while in the more distal conducting airways this function is taken over by club cells. The most distal component of the airways consists of the alveolar sacs, which is where gas exchange occurs. More than 95% of the alveolar surface is covered by very thin, flat alveolar type 1 (AT1) cells that allow for gas exchange. The remaining surface of the alveoli is composed of cuboidal alveolar type II (AT2) cells that play many important roles including surfactant production and serving as progenitors for AT1 cells (Barkauskas et al., 2013). It is important to note that there are some differences between the airways of humans and mice. One difference is that in humans, conducting bronchioles transition to respiratory bronchioles, which have alveolar outpouchings, before finally terminating in alveolar sacs, whereas in mice conducting bronchioles transition directly to alveoli at the bronchoalveolar duct junction (BADJ). Another major difference is that in uninjured mice basal cells do not extend past the most proximal bronchi, while in humans they can be seen to the level of the respiratory bronchiole (Basil et al., 2020). Therefore, it is worth taking these differences into consideration when comparing mouse and human studies.

The stem cell niche has been shown to be important for the maintenance of fundamental stem cell properties such as self-renewal and differentiation (Schofield, 1978; Jones and Wagers, 2008). Given that stem cells within a niche are responsive to signals sent from neighboring cells, the SASP is a potential modifier of stem cell function. Inflammatory cytokines (IL-1a, IL-1b, IL-6, MCP1), chemokines (CCL8), matrix metalloproteinases (MMPs), and growth factors (VEGF, EGF, EREG) are part of the SASP known to contribute to the maintenance of stem cells and their resident niches (Coppé et al., 2010; Kang et al., 2011; Reyes de Mochel et al., 2020). Notably, the SASP is both variable and dynamic, having been shown to exhibit significant changes during injury and regeneration. This responsiveness to the environment allows senescent cells to serve multiple functions depending on context (Krizhanovsky et al., 2008; Demaria et al., 2014). With respect to the lung, decades of research have highlighted the importance of the microenvironment in regulating lung stem and progenitor cell function. Furthermore, the identification of senescent cells in the lung stem cell niche could provide important insights in terms of how SASP derived from senescent niche cells could alter the regenerative response. Here are some examples of prominent SASP components that can influence stem cell behavior in the lung:

### Effects of IL-6 on Lung Stem Cells

In addition to its well-known role in regulating cells of the immune system, it is now appreciated that interleukin 6 (IL-6) can also modify lung stem cell behavior. Depending on the stimulus, IL-6 can be produced by a wide variety of cells including epithelial cells, macrophages, and fibroblasts (Crestani et al., 1994; Rincon and Irvin, 2012). After acute injury, airway basal cells are tasked with differentiating into secretory or ciliated cells to restore essential airway architecture (Rock et al., 2009). Tadokoro et al. demonstrated that after inhaled sulfur dioxide intratracheal injury, basal cells are stimulated with IL-6, which causes preferential differentiation of basal cells into ciliated cells. Interestingly, in this study fibroblasts were identified as the predominant source of IL-6, highlighting the role of stromal cells in supporting airway epithelial stem cell function after injury (Tadokoro et al., 2014).

The role of IL-6 in alveolar homeostasis and repair is less clear. In homeostasis, activation of the IL-6/STAT3 pathway in AT2 cells has been shown to be critical for proper lamellar body formation and surfactant production (Matsuzaki et al., 2008). After injury, IL-6 has been found to play either beneficial or detrimental roles dependent on both timing and injury. Using the bleomycin fibrotic lung injury model, it was shown that neutralizing IL-6 during the early inflammatory phase worsened fibrosis, while IL-6 neutralization during the subsequent fibrotic phase decreased fibrosis. In the setting of influenza, IL-6 has been shown to mediate phagocytic activity in macrophages, increase fibroblast apoptosis, and improve epithelial survival, thus promoting viral clearance while maintaining the epithelium and minimizing fibrosis. Accordingly, IL-6−/− mice experience worse disease severity after influenza compared to wild-type mice (Yang et al., 2017). This is also consistent with data showing that IL-6 secreted by alveolar mesenchymal niche cells can promote AT2 growth (Zepp et al., 2017). Taken together, it is clear that IL-6 can play many roles within the lung during homeostasis and injury. Future studies to better clarify the cellular targets and context-specific functions of IL-6 will be important for improved targeting of this important inflammatory pathway.

### Effects of IL1β/TNF on Lung Stem Cells

During lung homeostasis, AT2s serve as the resident stem cell in the main gas exchange compartment of the lung, maintaining alveolar epithelial populations through self-renewal and differentiation into AT1s (Barkauskas et al., 2013). In severe fibrotic injury, the lung develops alternate mechanisms to quickly regenerate AT2 and AT1 cells. AT2s can be regenerated from pre-existing AT2s or airway progenitors that migrate into the alveoli (Basil et al., 2020). Relevant to the mechanism of how airway progenitors differentiate into AT2s, a recent study showed that IL-1b secreted by infiltrating monocyte/macrophages regulates the airway progenitor niche to promote secretory cell transdifferentiation into AT2 cells (Choi et al., 2021). It has also been described that there is a subpopulation of AT2 cells that express the IL-1 receptor, *Il1r1*. In the setting of injury, IL-1b supports the development of a damage-associated transient progenitor (DATP) from these *Il1r1+* AT2 cells, an important intermediary step in the AT2 to AT1 transition. Interestingly, the downregulation of IL-1b signaling in DATPs is important for the completion of their transition to AT1 cells. In the setting of chronic inflammation, cells are stalled in the DATP state, impairing effective alveolar regeneration (Choi et al., 2020).

Another important characteristic of IL-1b signaling in the stem cell niche is that IL-1b can also directly activate the stromal niche that supports lung stem cells. AT2 organoids co-cultured with fibroblasts deficient for *Il1r1* displayed reduced organoid capacity when stimulated with IL-1b (Katsura et al., 2019). This is consistent with the study of DATPs that demonstrated the cell-autonomous requirement of *Il1r1* in AT2s to transition into AT1s, but not cell proliferation (Choi et al., 2020). These data suggest that IL-1b exerts antagonistic effects in the stem cell niche through direct activation of AT2s to promote differentiation, while indirectly promoting AT2 self-renewal by upregulating stem cell mitogens in AT2-supporting niche cells. Consistent with these data, we have recently demonstrated that IL-1b preferentially upregulates IL-6 and EREG in senescent fibroblasts to increase club cell proliferation (Reyes de Mochel et al., 2020).

Similar to IL-1b, TNF is another inflammatory cytokine that can activate NF-kB to promote SASP. Furthermore, TNF can induce a positive feedback loop by activating NF-kB to produce more TNF (Falvo et al., 2010). In the influenza lung injury model, 7 days after infection there is a surge of IL-1 and TNF in the damaged areas containing very few AT2 (SPC+) cells and within the injury perimeter. Like IL-1b, TNF was also shown to drive AT2 proliferation in the organoid assay *in vitro*. Additionally, mice deficient in *myd88*, an effector in the Toll-like receptor (TLR) pathway that can drive TNF production, demonstrates impaired AT2 repair after severe alveolar injury (Katsura et al., 2019). The source of TNF in these cases is yet to be determined, but likely candidates include recruited immune or resident mesenchymal cells. Although it is unclear if the origin of these cytokines is from senescent cells in the microenvironment, it is evident that common SASP factors produced in the niche can significantly impact resident stem cells.

### Effects of EREG on Lung Stem Cells

In our recent study, we found that EREG/Epiregulin is preferentially upregulated in *p16+* fibroblasts after epithelial injury. EREG was previously identified as a keratinocyte mitogen that acts through EGFR and ErbB4 (Draper et al., 2003). Genetic knockout of *Ereg* did not alter airway epithelial composition in healthy mice, but these animals failed to regenerate club cells after airway stem cell depletion. Interestingly, *Ereg* expression was rapidly induced in senescent fibroblasts in response to inflammatory stimuli such as LPS, IL-1b, or monocyte co-culture (Reyes de Mochel et al., 2020). This was fascinating because inflammatory signals accompany any tissue injury where the epithelial compartment is compromised. Our data demonstrate that senescent cells in the niche serve as a sentinel that senses inflammatory signals associated with injury to augment support for resident stem cell recovery.

The SASP appears to be modulating stem cell differentiation and renewal in a context-dependent manner. While senescent cells release the SASP to support growth, regeneration, or inflammation, there is reciprocal interaction with the microenvironment that feeds back to alter SASP components in senescent cells. The SASP appears to be dichotomous in that it increases epithelial plasticity/regeneration while increasing immune cell activation and recruitment. Unraveling what drives this contextual specificity may provide insight into how fluctuating SASP secretion predisposes the lung to age-related changes.

## Stem Cell Senescence in the Lung

Stem cells undergo age-related changes that alter function, but not necessarily with characteristics that are consistent with senescence. Muscle stem cells actually increase proliferation with age, but they lose but they lose differentiation capacity and differentiation capacity that can be attributed to alterations in the aging muscle stem cell niche (Chakkalakal et al., 2012). Much less is known about how lung stem cell function changes with age, or whether senescence is a feature of lung stem cell aging. What is apparent is that recent literature has highlighted transitional cell states in lung stem cells that approximate certain features of senescence. Single-cell analyses of injured AT2s *in vivo* and *in vitro* have recently shown transient intermediates that appear during the differentiation of AT2s to AT1s. These transitional cells are known as pre-alveolar type I transitional cell state (PAT), alveolar differentiating intermediate (ADI), or damage-associated transition progenitors (DATPs) (Choi et al., 2020; Jiang et al., 2020; Kobayashi et al., 2020; Strunz et al., 2020). Despite the varying nomenclature, all the pre-AT1 intermediates described have similar low AT2 and high AT1 transcriptional profiles with high Keratin 8+ expression (Choi et al., 2020; Kobayashi et al., 2020; Strunz et al., 2020). Interestingly, the AT2 to AT1 intermediates morphologically change from a cuboidal to a flattened extended morphology, a characteristic of senescent cells. Furthermore, transcriptome analysis indicates an upregulation of genes associated with senescence such as *p21*, *p16^INK4a^*, and *p53* in the transitional cell types. While it remains unclear if these cells are truly “senescent,” these transitional cells display features of senescence, including activation of NF-kB, TP53, and DNA damage response pathways as they undergo differentiation into AT1s. It will be important in future studies to further clarify the role the senescent program plays in the formation of these transitional states, although existing data suggest that IL-1b and TGFb are likely important contributors (Choi et al., 2020; Kathiriya et al., 2022). Furthermore, there is evidence that these transitional cell states are more prevalent in age-related lung diseases (reviewed below), however, it is not clear whether they are associated with normal aging in the human lung.

## Changes in Lung Cellular Composition and Function With Age

### Parenchymal Changes in the Lung

Alterations in both airway cellular composition and function have been observed with age, and growing evidence points to cellular senescence as a contributor to these changes. In the conducting airways, several studies have identified a decrease in airway epithelial density with age, as well as a decrease in the ratio of epithelial progenitor cells relative to their ciliated counterparts (Wansleeben et al., 2014; Ortega-Martínez et al., 2016; Angelidis et al., 2019). Given the known effect IL-6 plays in skewing basal cells toward a ciliated identity, it is tempting to speculate that aging-associated increases in local IL-6, either from senescent stromal cells or resident immune cells, may underlie these compositional changes (Tadokoro et al., 2014; Wansleeben et al., 2014). To date, the functional significance of these epithelial alterations remains unclear, as there appears to be no age-related impairment of tracheal epithelial repair after inhalation of sulfur dioxide (Wansleeben et al., 2014). However, one possibility is that this age-associated increase in the proportion of ciliated cells is a compensatory mechanism for impaired ciliary cell function (Bailey et al., 2014). Indeed, in aged ciliated cells, oxidative stress, which has been linked to both aging and senescence, has been shown to be responsible for decreased ciliary beat frequency, thus providing a direct mechanism by which aging impairs mucociliary clearance (Takahashi et al., 2006; Bailey et al., 2018). It is likely that this age-associated impairment in mucociliary clearance contributes to the increased risk of severe pneumonia observed among the elderly (Schneider et al., 2021).

In the alveolar compartment, airspace enlargement is one of the hallmark changes of lung aging. Alveolar enlargement, accompanied by an increase in lung compliance and decrease in lung elasticity, is commonly referred to as “senile emphysema” (Janssens et al., 1999; Cho and Stout-Delgado, 2020). One proposed mechanism for this finding is impaired AT2 to AT1 differentiation with age, leading to a decreased total number of AT1 cells (Schneider et al., 2021). This finding is supported by the observation that while there are consistent levels of EdU incorporation within AT2 cells of young and aged mice, in aged mice there is a decrease in the number of EdU positive AT1 cells, the terminally differentiated product of AT2 cells (Watson et al., 2020). This mechanism would be consistent with many other studies that have demonstrated age-related alterations in stem cell differentiation capacity (Schultz and Sinclair, 2016). Impaired AT2 function is likely attributable to both cell-intrinsic and cell-extrinsic changes that occur with age. For example, it has been shown that AT2 cells from aged mice have increased features of senescence and an impaired ability to form alveolar organoids *in vitro*, demonstrating cell-intrinsic changes with age (Lehmann et al., 2020). Additionally, an increase in oxidative stress within the alveolar epithelium preceding emphysema formation has been observed, offering a potential underlying mechanism for this AT2 dysfunction (Calvi et al., 2011). However, it has also been shown that lung stromal cells from aged mice exhibit several senescent cell characteristics in addition to increased *Nox4* expression, thus enhancing ROS production. *Nox4* deletion within aged stromal cells was able to partially restore alveolar organoid formation *in vitro*, demonstrating a role for senescent stromal cells in modulating AT2 cell function, and potentially even contributing to AT2 aging-induced senescence through ROS production (Chanda et al., 2021). Indeed, antioxidants have been shown to both increase the survival and modify the proliferative and differentiation capacity of stem cells, raising the possibility of antioxidants as therapy for aging-associated lung dysfunction (Abdel-Daim et al., 2017).

Interestingly, in addition to ROS, an increase in the number of lung macrophages and B cells has been shown to coincide with the timing of aging-associated airway enlargement (Calvi et al., 2011). As discussed above, chronic IL-1b treatment of AT2 cells leads to the persistence of DATPs and impaired AT1 differentiation (Choi et al., 2020). Given the previously identified role for macrophage IL-1b in the formation of DATPs, it is possible that IL-1b or other inflammatory cytokines released by the increased number of macrophages within the lungs of aging mice contribute to the observed impairment of AT2 to AT1 differentiation, although to our knowledge this hypothesis has not yet been formally tested.

A direct role for senescent cells in mediating age-related parenchymal changes was identified using an ARF-diphtheria toxin receptor (DTR)-luciferase transgenic mouse to both track and inducibly delete *p19* expressing cells. Using this system, it was observed that there is significant upregulation of *p19*, as well as other senescence-associated genes such as *p16* ^*INK4a*^ and *p21* in the lungs of healthy aged mice, with the majority of *p19* expression attributable to lung fibroblasts. Interestingly, removal of *p19* cells with the use of diphtheria toxin decreased age-associated increases in lung compliance and improved tissue elastance (Hashimoto et al., 2016). Given the well-appreciated role of senescent cells in MMP production, one can postulate that in addition to altering the epithelial compartment, senescent lung fibroblasts may contribute to the ECM remodeling and changes in collagen seen with age (Muñoz-Espín and Serrano, 2014; Tabula Muris, 2020).

Lastly, while not historically considered a prominent feature of lung aging, there is mounting evidence that fibrosis may play a role in this process. With the increased use of chest computed tomography (CT) for screening and diagnostic purposes, it is becoming apparent that many clinically healthy older individuals have radiographically appreciable interstitial abnormalities (Hatabu et al., 2020). Supporting this observation, a recent study analyzed lung tissue from 86 deceased donors between the ages of 16 and 76 without known lung pathology and found that with age there was an increase in the expression of collagen processing genes and histological evidence of subpleural fibrosis in those of advanced age. They also note that the observed age-associated changes in pro-fibrotic gene expression positively correlate with signs of cellular senescence, including *CDKN2A* expression, shortened telomeres, and gamma-H2AX foci (Lee et al., 2021). As discussed later, senescence has been linked to the pathophysiology of the age-associated disease idiopathic pulmonary fibrosis (IPF). It will be interesting to determine whether the same senescent pathways are involved in fibrosis that develops during normal aging, and if so, to what extent IPF represents an accelerated aging phenotype.

### Changes in the Lung Immune System

Aging is accompanied by significant alteration in both the innate and adaptive immune system that leads to a state of immune system dysfunction and impaired response to pathogens and vaccines. Together, these changes are commonly referred to as immunosenescence (Oxman et al., 2005; Nikolich-Žugich, 2018). As an example, dendritic cells, which are critical for activating and instructing the adaptive immune system, have impaired maturation, migration, and ability to cross-present antigens with age (Panda et al., 2010; Zhao et al., 2011; Zacca et al., 2015). Additionally, cell-intrinsic changes in aged hematopoietic stem cells (HSCs) skew their differentiation potential to a myeloid rather than lymphoid fate (Rossi et al., 2005). This altered differentiation potential contributes to a decrease in naive T cell production with age, and in conjunction with impaired naive T cell maintenance, leads to an overall decline in naive T cell numbers and diversity with age (Rudd et al., 2011). B cells are also affected by aging, demonstrating impaired immunoglobulin class switch recombination and somatic hypermutation, leading to less effective antibody responses in the elderly (Frasca et al., 2004). While immune cell senescence has been well-documented, it remains to be determined to what extent most age-related changes in the immune system are attributable to cellular senescence versus other age-associated alterations in function (Brenchley et al., 2003; Wherry, 2011; Crespo et al., 2013).

Another concept that has become prominent in the aging literature is that of “inflammaging”, referring to the development of a state of chronic low-grade inflammation with age (Franceschi et al., 2018). There is some data to support this notion. For example, dendritic cells isolated from the circulation of aged individuals have been shown to have increased basal levels of proinflammatory cytokine production, despite having impaired cytokine response to TLR ligands (Panda et al., 2010). Additionally, several studies have demonstrated an increase in circulating pro-inflammatory factors, such as IL-6, IL-18, and C-reactive protein (CRP) with age, although these correlations become much weaker when underlying cardiovascular disease is taken into account, raising the question of how much these findings are intrinsic to the normal aging process (Ferrucci et al., 2005; Puzianowska-Kuźnicka et al., 2016). In humans, the pulmonary immune system is most easily evaluated with bronchoalveolar lavage (BAL), a technique that samples cells residing within the most distal airways. In healthy individuals, the vast majority of BAL cells are alveolar macrophages, although this has been shown to change with age. In a study of clinically healthy, never-smokers over a spectrum of ages, individuals in the oldest group had increased neutrophils, elevated levels of BAL IL-6, and increased BAL superoxide anion production in response to PMA, all consistent with a basal inflammatory state (Meyer et al., 1996). While there are marked changes in the immune system with age, much more needs to be done to determine the extent to which healthy aging is accompanied by an increase in basal inflammation, as well as elucidating the sources and functional consequences of these secreted inflammatory mediators.

It is likely that alterations in the immune system play a role in lung aging, and that age-associated changes in the immune system are both cell intrinsic and in response to an aging microenvironment. The contribution of immune cell senescence to driving solid organ aging was recently tested using a genetic model to specifically suppress DNA damage repair within the hematopoietic system. Using this model, the authors were able to demonstrate that immune cell senescence not only phenocopied many aging-associated physiologic changes but also induced features of senescence in parenchymal tissues, including the airway epithelium (Yousefzadeh et al., 2021).

Conversely, there are multiple examples by which aged parenchymal cells alter the immune system. For example, in the setting of influenza, aged mice accumulate more neutrophils in their BAL compared with young mice. Depletion of neutrophils after infection dramatically improved survival in aged mice, while there was not a significant improvement in survival among young mice, demonstrating the role of excessive neutrophilic inflammation in influenza mortality. Importantly, it was found that increased neutrophil chemotaxis to the lungs in aged mice was attributable to elevated secretion of the neutrophil chemoattractants CXCL1 and CXCL2 from airway epithelial cells that exhibited increased expression of the senescent marker SA-beta-galactosidase (Kulkarni et al., 2019). As discussed previously, the ability of senescent cells to recruit immune cells for the purpose of immunosurveillance is a core function of the SASP. This study demonstrates how the accumulation of cells with senescent features may prime tissues for an exaggerated inflammatory response and inadvertent immunopathology in the setting of injury.

There is also growing evidence that in addition to increasing immune cell recruitment, senescent cells or other alterations in the aged microenvironment can modify immune cell phenotype. A recent study evaluated changes in alveolar macrophages (AMs) with age, and identified that AMs are decreased in number in aged mice, have impaired proliferation in response to GM-CSF, and exhibit a pro-inflammatory gene expression profile. Notably, this phenotype was largely determined by the aged lung microenvironment, as heterochronic transfer of aged AMs to young mice restored AM gene expression to that of young AMs. The authors went on to show that hyaluronan inhibits macrophage proliferation, and argue that increased hyaluronan production by aged AT2 cells may be partially responsible for the effect of the aged lung microenvironment on AMs (McQuattie-Pimentel et al., 2021). Results from another recent study comparing scRNA sequencing of immune cells from young and aged mice describe the development of a unique granzyme K (GZMK) positive CD8^+^ T cell population with age, which they call T aging-associated (Taa). Notably, the formation of this CD8^+^ Taa population was dependent on the aged microenvironment, although the specific factors driving CD8^+^ Taa development remain unknown. They also show that GZMK is able to enhance SASP secretion from senescent cells, raising the possibility of an inflammatory positive feedback loop between senescent cells and the immune system (Mogilenko et al., 2021).

**FIGURE 1:**
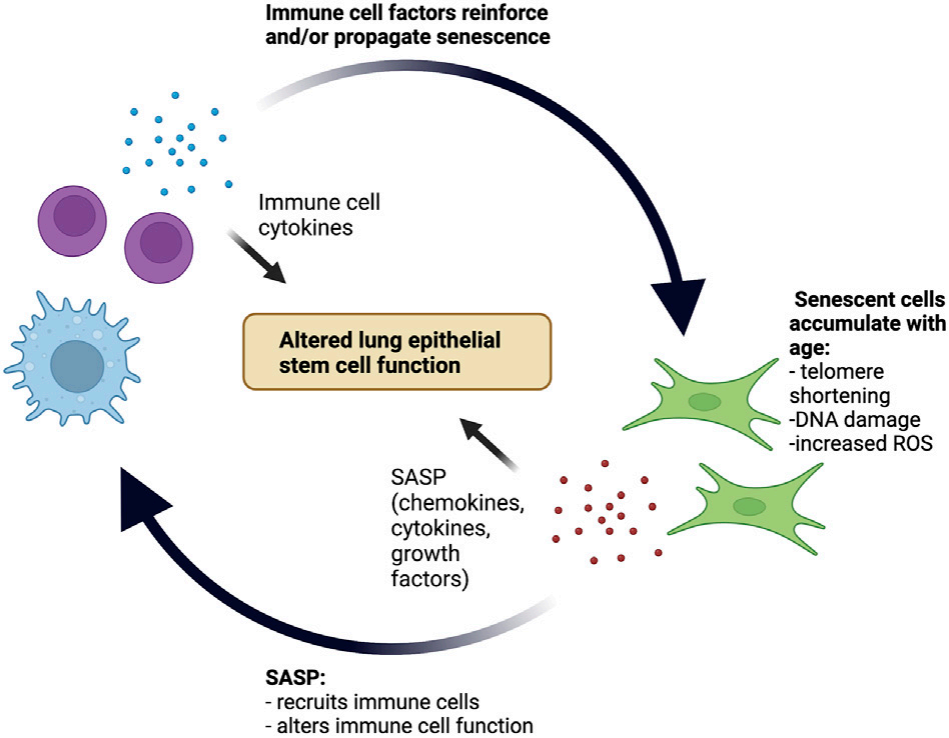
Positive inflammatory feedback loop between senescent cells and the immune system in the aging lung stem cell niche. With age, senescent cells accumulate within the lung parenchyma and secrete SASP factors that both recruit immune cells and alter immune cell function. Subsequently, recruited immune cells secrete cytokines that reinforce and potentially even propagate cellular senescence. The pro-inflammatory environment created by this positive feedback loop contributes to age-related alterations in lung epithelial stem cell function.

Together, emerging data support a scenario in which the aging lung parenchyma and immune system are in close interaction. Senescent parenchymal cells can modify both the composition and phenotype of the resident immune cell compartment. Similarly, recruited aged and senescent immune cells reciprocate by driving the propagation of senescence within the parenchyma. The sum of these interactions is the development of an inflammatory positive feedback loop, creating chronic low-grade inflammation with age that not only alters tissue function in homeostasis but sets the stage for a dysregulated response to future insults (Figure 1).

## Evidence of Senescence in Aging-Associated Lung Disease

Age is one of the strongest risk factors for some of the most prevalent chronic lung diseases, including emphysema, lung fibrosis, and lung cancer (Rojas et al., 2015). There is growing evidence that physiologic aging in the lung can underpin the susceptibility to these chronic lung diseases. The following section discusses the evidence that senescence plays a role in these age-related lung diseases.

### Emphysema

Emphysema is a condition characterized by alveolar wall destruction with associated airspace enlargement. It is part of a larger group of lung impairments called chronic obstructive pulmonary disease (COPD), a chronic inflammatory lung disease that leads to airflow obstruction and difficulty breathing (Celli and Wedzicha, 2019). It has been noted that multiple cell lineages, including epithelial, endothelial, and fibroblasts from patients with COPD have increased features of senescence, including DNA damage, *p16^INK4a^* expression, and the development of a SASP (Tsuji et al., 2006; Woldhuis et al., 2021). Cigarette smoke, one of the strongest risk factors for emphysema, has been shown to induce senescence in lung fibroblasts (Nyunoya et al., 2006). Additionally, the genetic elimination of *p19*ARF-expressing cells was able to reduce both emphysema formation and immune cell infiltration in a murine model of emphysema (Mikawa et al., 2018).

Short telomere length is thought to be one of the major drivers of cellular senescence in COPD, as individuals with COPD have been found to have decreased telomere length compared to healthy controls (Mui et al., 2009). Additionally, mice with short telomeres have accelerated development of emphysema in the setting of chronic cigarette smoke exposure (Alder et al., 2011). Activation of the mTOR pathway is another mechanism by which cellular senescence may develop in emphysema. The mTOR pathway, a known inducer of senescence, displays increased activation in patients with COPD relative to controls, and *in vitro* inhibition of the pathway with rapamycin decreased cellular senescence in pulmonary endothelial and smooth muscle cells from patients with COPD (Houssaini et al., 2018). Furthermore, activation of the mTOR pathway through TSC1 deletion in either endothelial or AT2 cells was sufficient to drive the development of emphysema in mice (Houssaini et al., 2018). Similarly, inhibition of the mTOR pathway with the AMP-activated protein kinase (AMPK) activator metformin was shown to decrease the development of senescence, SASP secretion, and emphysema in the elastase-induced murine model of emphysema (Cheng et al., 2017). Taken together, current evidence suggests that cellular senescence contributes to the pathogenesis of emphysema, and raises the potential for senolytics in the treatment of this disease.

### Idiopathic Pulmonary Fibrosis

Idiopathic pulmonary fibrosis (IPF) is a chronic, progressive form of pulmonary fibrosis of unknown cause. It is a disease of aging, most commonly diagnosed in the sixth or seventh decade of life, and rarely appreciated before the age of 50 years (Lederer and Martinez, 2018). A causal link to the age-related cellular process was established when it was discovered that mutations in the telomerase genes *hTERT and hTR* are a significant contributor to familial pulmonary fibrosis (Armanios et al., 2007). Further supporting this connection is that many individuals with sporadic IPF have also been found to have short telomeres (Alder et al., 2008). Mechanistic studies in mice demonstrated that deletion of the telomere shelterin gene, *Trf1,* induced features of senescence in AT2s and increased lung fibrosis with age (Naikawadi et al., 2016). Additionally, a mouse model in which p53 was specifically activated in AT2 cells was sufficient to induce senescent features in AT2s and drive progressive lung fibrosis (Yao et al., 2021). In IPF lungs, numerous cell types that bear resemblance to transition cell types in mice have also been identified, with similar upregulation of senescent gene programs (Adams et al., 2020; Habermann et al., 2020; Kobayashi et al., 2020). This raises the possibility that the senescent gene program might play a role in the stem cell metaplasia commonly seen in fibrotic lung remodeling, where ectopic airway basal cells appear in the alveoli in abnormal cystic structures (Lederer and Martinez, 2018). Our group has recently shown that human AT2s can differentiate into metaplastic basal cells in culture through transitional states that resemble those found in IPF, and these transitional states also upregulate similar senescence-associated genes (Kathiriya et al., 2022).

Fibroblast senescence has also been shown to play a role in lung fibrosis. Aged mice, in contrast to their young counterparts, develop persistence of lung fibrosis after bleomycin injury. This phenotype was shown to be attributable to the persistence of senescent fibroblasts and ROS production in aged mice through an imbalance between the ROS-producing enzyme Nox4 and the antioxidant protein Nrf2. Interestingly, they show that fibroblasts from IPF lungs also have increased levels of Nox4 protein, suggesting that a similar impairment in the regulation of ROS may be at play in IPF pathogenesis (Hecker et al., 2014). A subsequent study showed that depletion of *p16* ^*INK4a*^ *+* cells improved lung mechanics after bleomycin injury (Schafer et al., 2017). A recent single-cell atlas of IPF also demonstrated upregulation of *CDKN2A* (gene encoding p16) in a subset of fibroblasts that arise *de novo* in IPF (Habermann et al., 2020). Together, these studies support a causative role of cellular senescence in IPF pathogenesis, which has prompted clinical investigation into senolytics as a therapeutic for fibrotic lung disease (Justice et al., 2019).

### Lung Cancer

At first glance, it would appear that senescence has a clear role in suppressing lung tumor formation and progression. This is supported by data showing that a large percentage of non-small cell lung cancers (NSCLC) demonstrate methylation-induced silencing at the *p16/CDKN2A* locus (Sterlacci et al., 2011). However, this has not been supported in studies where p16 is correlated with outcome, with some studies showing that p16 + cells on histology or *p16* transcripts directly correlate with worse survival from lung cancer (Győrffy et al., 2013; Pezzuto et al., 2020).

Supporting a pro-tumorigenic role for cellular senescence, it has been reported that senescent cells provide a milieu to support cancer cell proliferation *in vitro* that is predominantly mediated by the SASP (Krtolica et al., 2001). *In vivo*, it has been shown that *p16* expression increases in both the neoplastic and surrounding stromal cells early in tumor development, including in a model of lung adenocarcinoma (Burd et al., 2013). Using *in vivo* tumor models, SASP factors have been found to promote various aspects of tumorigenesis including metastasis and angiogenesis in addition to proliferation. For example, the IL-6-activated STAT3 pathway can lead to an increase in lung cancer cell growth (Song et al., 2011). Additionally, several studies have shown that senescent cells can support the proliferation of tumor cells when co-injected into immunocompromised mice (Coppé et al., 2006; Liu and Hornsby, 2007). Drawing a parallel to our prior study showing that senescent niche cells can support airway stem cell growth (Reyes de Mochel et al., 2020), these data suggest that senescent niche cells in the tumor stroma can also provide positive signals to tumor stem cell subsets that might be responsive to SASP.

Notably, the induction of cellular senescence in stromal cells as an unintended consequence of cancer therapy is starting to receive increased attention due to the increased number of cancer survivors (Zhu et al., 2020). A study showed that *p16^INK4A^* is upregulated in stromal cells after chemotherapy treatment, and the presence of these cells increased tumor progression and metastasis in a genetic model of breast cancer metastases (Demaria et al., 2017). Considering that many cancer therapeutics promote senescence and SASP, targeting senescent cells in combination with conventional therapeutic approaches is a potential way to improve therapeutic effectiveness and decrease recurrence rates. For example, the FDA-approved histone deacetylase inhibitor, panobinostat, was able to promote senolysis of chemotherapy-induced persistent senescent cells in non-small cell lung cancer (NSCLC). Administration of panobinostat after Taxol treatment in a non-small cell lung cancer cell line decreased Bcl-xL expressing persistent senescent cells (Samaraweera et al., 2017). These studies suggest that monitoring for the emergence and persistence of senescent cells after cancer treatments may be helpful in predicting the risk of tumor relapse in lung cancer patients.

## Conclusion

As the population ages, it is becoming increasingly important to understand aging-associated diseases and how physiologic aging increases susceptibility to these conditions. The lung is of particular significance in this context given the large extent to which aging-associated lung disease contributes to morbidity and mortality within our societies. There are many lines of evidence supporting a role for senescent cells in both physiologic lung aging and aging-associated lung diseases, making the prospect of senolytics for therapeutic purposes very appealing. However, the fundamental function of cellular senescence as a tumor-suppressive mechanism cannot be ignored, and the growing evidence for a positive role of senescence in the context of acute injury demonstrates the importance of increasing our understanding of these complex cells before indiscriminately eliminating them. Similarly, it is becoming clear that the development of senescence is a dynamic and ongoing process, making improved understanding of senescent cell heterogeneity critical for more targeted therapies.

It is now clear that senescence and inflammation are intertwined. Inflammatory signals from senescent cells are important not only for induction of immunosurveillance, but also for maintenance of their own senescent identity. What is less understood is how inflammation mediated by the SASP affects tissue and stem cell function, and to what extent these effects are context dependent. Many studies suggest senescent cells impair tissue homeostasis and stem cell function. Conversely, it has been shown that inflammatory cytokines, including the common SASP factors IL-1b and IL-6, play an important function in promoting airway epithelial repair. How then can one reconcile these seemingly opposing roles? One potential explanation may be differences in the duration of inflammatory signals, in which short-term inflammation in the setting of acute injury is important for regeneration, whereas prolonged inflammatory signaling from senescent cells leads to stem cell exhaustion or other dysfunction. Further studies to better understand how SASP factors alter stem cell function both in homeostasis and after injury will be important for improving tissue regeneration in the elderly. Finally, there is growing evidence that in addition to simply recruiting immune cells, senescent cells and the aged microenvironment can alter immune cell function. This is an exciting area of research and raises the possibility that changing the microenvironment may be able to slow or even reverse the decline in immune function that occurs with age.
